# Facial Expressions as a Nexus for Health Assessment

**DOI:** 10.3390/bioengineering13020208

**Published:** 2026-02-12

**Authors:** Jinani Sooriyaarachchi, Di Jiang

**Affiliations:** Medical Devices Research Centre, National Research Council of Canada, Boucherville, QC J4B 6Y4, Canada

**Keywords:** computer vision, facial expressions, remote biosensing, health assessment, pain, cognitive impairments

## Abstract

Facial expressions are crucial in conveying emotions and for engaging in social interactions. The facial musculature activations and their pattern of movements under emotions are similar in all humans; hence, facial expressions are considered a behavioral phenotype. Facial features related to the expression of various emotions change under different health impairments, including cognitive decline and pain experience. Hence, evaluating these facial expression deviations in comparison to healthy baseline conditions can help in the early detection of health impairments. Recent advances in machine learning and computer vision have introduced a multitude of tools for extracting human facial features, and researchers have explored the application of these tools in early screening and detection of different health conditions. Advances in these studies can especially help in telemedicine applications and in remote patient monitoring, potentially reducing the current excessive demand on the healthcare system. In addition, once developed, these technologies can assist healthcare professionals in emergency room triage, early diagnosis, and treatment. The aim of the present review is to discuss the available tools that can objectively measure facial features and to record the studies that use these tools in various health assessments. Our findings indicate that analyzing facial expressions for the detection of multiple health impairments is indeed feasible. However, for these technologies to achieve reliable real-world deployment, they must incorporate disease-specific facial features and address existing limitations, including concerns related to patient privacy.

## 1. Introduction

As social creatures, humans engage in a multitude of daily interactions and communications through the exchange of information. Facial expressions play a pivotal role in sending and receiving messages during these interactions, and are evoked by specific facial muscle movements to act as powerful non-verbal cues in communication, such as non-verbal emotions. Face-to-face interactions occur from the beginning of existence between parents and infants and are important for parents to evaluate the affective state of their infants, whose verbal communication channels have not yet developed [1]. Often, observing the entire face is not necessary to understand the emotions conveyed through facial expressions; individual facial regions are adequate to detect emotions, for example, sad or angry eyes and a happy mouth [2].

Facial expressions can be controlled voluntarily. The communicator can suppress or conceal the actual emotion to show a different emotion, or involuntary expressions, which can reveal the genuine emotion in the form of subtle micro-expressions [3]. Studies suggest that facial expressions are a behavioral phenotype, which, under similar circumstances, activate the same facial muscles and facial movements [4]. This universal similarity of facial expressions has contributed vastly to social intelligence and efficient social interactions. However, the expression, perception, and interpretation of facial expressions can be altered under various disease conditions. Therefore, tracking these facial expression deviations from the baseline conditions can be used as an objective tool in health assessment, especially in conditions where the patient loses the ability to verbally convey the disease or the pain associated with it.

This review focuses on the feasibility and current research on the use of facial expression cues and facial features in different health assessments. Although previous reviews have reported facial expression-based studies related to a specific disease condition [5,6,7,8], to the best of our knowledge, this is the first review article that carries out a cross-condition comparison. This present review summarizes several widely used tools available for assessing facial expression frequencies and their magnitudes, discusses a broad range of applications of monitoring facial expressions across several health impairments, and finally, evaluates the advantages and limitations of this approach. Furthermore, based on existing research, we highlight the need for condition-specific facial expression-based detection models that incorporate disease-specific activation of facial features.

## 2. Tools for Objective Measurement of Facial Expressions

### 2.1. Facial Action Coding System (FACS)

Originally developed by psychologists Paul Ekman and Wallace Friesen in 1978 [9], the Facial Action Coding System (FACS) identifies a standard set of facial muscle movements corresponding to different facial expressions. FACS combines the activation and relaxation of 46 facial muscles known as action units (AUs) to objectively measure the changes in facial expressions. For example, AU6 and AU12, respectively, represent the activation of Orbicularis oculi (check raiser) and Zygomatic Major (lip corner puller) facial muscles, both of which are activated in happiness and joy. Alternatively, the combined activation of the levator labii superioris alaquae nasi (nose wrinkler), depressor anguli oris (lip corner depressor), and depressor labii inferioris (lower lip depressor) muscles is associated with facial expression displaying disgust. In FACS, the activation intensity of AUs is indicated using a scale between 0 (complete relaxation) and 5 (complete activation).

Due to its objective facial expression measurement capabilities and the capability of supporting high temporal resolution facial videos, which allows the study of subtle micro-expressions, FACS is widely used in fields such as human–computer interaction and psychology to analyze human behavior, social interactions, and evoked emotions in response to triggers and stimuli. Although in the past the accuracy of FACS was dependent on training experienced FACS coders and their inter-individual perceptual differences, modern machine learning-based automated FACS tools such as OpenFace [10], Py-Feat [11], Noldus FaceReader [12], and Affdex [13] provide efficient measurements of AU activations and their intensities and facial expressions without manual intervention. However, the predictive performance of these automated tools can vary based on multiple factors, including pose, occlusion, and face angle [14]. Therefore, it is important to consider the performance, as well as licensing constraints and validation contexts, when choosing these tools in future research.

### 2.2. Facial Electromyography (EMG)

Facial electromyography (EMG) provides an objective method to record the activity of facial muscles with a high temporal resolution and sensitivity; therefore, it is capable of capturing the slightest muscle contractions that might not be visible to the naked eye. The discovery of EMG is a result of the consecutive work of multiple neurophysiologists, from the concept of animal electricity (i.e., animal muscle contractions due to electrical stimulations) in 1771 to the invention of techniques to amplify bio-electrical signals at the beginning of the 20th century [15]. EMG activity, which can be recorded using surface electrodes, consists of the electrical signals related to muscle fiber depolarizations that evoke action potentials, causing muscle contractions [16]. Relaxation and contraction of facial muscles during different facial expressions can, therefore, be captured using facial surface EMG electrodes.

Despite many advantages of facial EMG, it carries several limitations, including the requirement of specialized equipment for data collection, higher inter-subject signal variability, and the electrode placements visually obscuring the face, making it less applicable in social contexts. However, the use of EMG to analyze facial expressions and emotions dates back to the 1970s. Based on recorded facial EMG activity from corrugator and zygomaticus muscle sites, researchers were able to differentiate and quantify the intensity of happy and sad emotions imagined by human subjects [17,18]. Recently, studies in EMG-based emotion recognition applying machine learning techniques with facial EMG features have achieved higher accuracies in distinguishing between multiple emotions [19,20]. Hence, the latest models of virtual reality headsets have started integrating EMG sensors for emotion detection during VR simulations [21].

### 2.3. Computer-Vision-Based Techniques

Computer vision, a branch of artificial intelligence and computer science that trains computers to understand and derive meaningful information from visual data inputs, including images and videos, has shown ground-breaking advances in recent years. It has introduced several techniques, including Face Mesh and Histogram of Oriented Gradients (HOG), that are used to extract facial features. Face mesh represents the three-dimensional geometry of the face, consisting of a collection of interconnected points arranged in a mesh-like pattern and interconnected vertices to approximate the shape of the face [22]. Recent advances in computer vision and machine learning have introduced several software tools to automatically extract the face mesh using images and videos, for example, MediaPipe, which computes a face mesh consisting of 478 vertices located on the key points on the human face [23]. Tracking the face mesh can be used to capture facial expressions [24] without capturing personal identity, thus protecting human privacy.

Histogram of Oriented Gradients (HOG) has been widely used to detect objects in computer vision applications [25]. It computes the gradient in small cells of an image, providing a compact representation of an object. Hence, HOG can be used to model the shape of facial muscles using edge analysis. HOG features have been effectively used in multiple facial expression recognition studies along with machine learning techniques [26]. HOG descriptors are capable of extracting features without being affected by illumination changes, proving this technique to be an added value. Various automated software tools are available to compute HOG, including OpenFace [10], which also simultaneously computes other facial features, including AUs, facial landmarks, head pose, and gaze tracking.

## 3. Facial Expressions in Health Assessment

For this narrative review, we conducted a literature search in the PubMed and Google Scholar databases (accessed July–August 2025) using a keyword-based approach (e.g., facial expressions, facial features, health detection). We included articles published after 1990, related to human facial expressions, and the articles that clearly described the deviations in facial expressions/features associated with different health conditions, as well as studies that used these deviations in the detection of the related health condition. These studies will be discussed below.

Various health impairments, including cognitive decline, pain experience, stroke, inflammation, migraines, and non-psychotic disorders, can cause deviations in the facial expressions of patients compared to healthy controls. Hence, recent years have witnessed an increasing interest and advancements in medical assessments using the aforementioned techniques and tools, especially in the initial screening phase and in remote settings, including telemedicine applications. Multiple studies have focused on the application of facial expressions and facial features-based technologies for the early detection of various health conditions. These will be discussed below in three main application sections: 1. detection of cognitive impairments, 2. detection of pain, and 3. detection of other health conditions.

### 3.1. Facial Expressions in Cognitive Impairments

Cognitive impairments, which can be mild or severe, affect a person’s cognitive abilities and daily functioning, such as thinking, memory, learning, and decision making. This has become a common condition among older adults. For example, in Canada, over 770,000 individuals were living with Alzheimer’s disease (AD) or other forms of dementia in 2025, and it is projected to reach 1.7 million by 2050. Dementia is a clinical diagnosis represented by the basis of progressive cognitive decline, and it can occur due to different pathophysiological processes, including AD (50–75%), vascular dementia (20%), dementia with Lewy bodies (5%), and frontotemporal lobar dementia (5%) [27]. AD, which is the most prevalent type of dementia, is named after the German psychiatrist Alois Alzheimer, who observed a massive loss of neurons in the cerebral cortex of his patient suffering from memory loss [28]. The state between normal aging and dementia, known as mild cognitive impairment (MCI), progresses to dementia at an annual rate between 8 And 15% and subsequently to AD [29]. Therefore, early screening for cognitive decline has become increasingly important.

Conventional cognitive screening methods, such as brain scans (CT, MRI, PET), are used widely to study early structural and metabolic changes in multiple brain areas, including the hippocampus, entorhinal cortex, and gray matter in the medial temporal lobe [30]. However, brain imaging tends to be resource-intensive, costly, and stressful for patients. Apart from brain scans, there exist multiple psychiatric tests to conduct cognitive assessment, such as the Mini-Mental Status Exam (MMSE) or Montreal Cognitive Assessment (MoCA). MMSE measures five areas of cognitive function, including orientation, registration, attention and calculation, recall, and language [31]. In comparison, MoCA covers more domains, including visuospatial skills, attention, language, abstract reasoning, delayed recall, executive function, and orientation, with a higher specificity and sensitivity [32]. However, these tests have statistical limitations; for example, they might not be strong enough to detect mild cognitive decline and might have a ceiling effect with high IQ patients [33]. Therefore, developing efficient and accessible alternatives to cognitive assessment is crucial.

The study of facial expression deviations during cognitive decline has emerged as an interesting technique for cognitive impairment detection. Previous research in this field can be broadly divided into two main areas: (1) exploratory studies that examine changes in facial expressions among cognitively impaired patients compared to healthy individuals, and (2) studies that assess facial expressions and features as a detection tool for distinguishing cognitively impaired patients from healthy controls.

#### 3.1.1. Exploratory Studies

There is a vast majority of research studying the facial expressions of cognitively impaired patients dating back to the 1990s. Asplund et al. (1991). who studied the ability of facial expression exhibition in four Alzheimer’s type dementia patients using FACS, found that these patients show decreased complex facial expressions associated with emotions under pleasant and unpleasant stimulus conditions [34]. This phenomenon, known as hypomimia (i.e., reduced facial expressions and facial cues), was reported in subsequent studies of severely demented patients using FACS, with facial videos recorded during caregiving activities [35]. A separate study with AD patients and Parkinson’s disease (PD) patients videotaped during neutral and posed facial emotions assessed using MDS-UPDRS 3.2 score (scale for facial expression ranging from score 0 (“normal facial mimic”) to score 4 (“Fixed facial expressions with lips open most of the time when the mouth is still”)) [36] showed similar patterns of reduced facial expressions in AD and PD compared to healthy controls [37].

Contrary to the concept of hypomimia in cognitive impairments, Seidl et al. (2012) showed that cognitive deficits are associated with an increased rate of total facial expressions. This was observed in recorded facial videos of AD patients, analyzed using emotional FACS (EMFACS) after controlling for apathy, in response to emotion-eliciting or neutral images [38]. A study conducted with dementia of Alzheimer’s type showed an increase in facial expressions associated with negative affect in reaction to sad vignette stimuli compared to the control group. These facial videos were also scored for facial expressions using the FACS [39]. Additionally, a facial EMG signal-based study conducted with AD subjects observed inverted patterns of zygomatic activity in comparison to healthy controls in response to emotion-eliciting images [40].

The aforementioned studies demonstrated that no consensus has been achieved that AD or dementia patients universally show reduced or increased facial expressions. Instead, the literature suggests a spectrum of changes. For some individuals in some contexts, they may show reduced emotional expressivity or flattened affect, and others may show increased facial expressions, which can be caused by various pathological reasons, such as less control over facial muscles, disinhibition, or emotional dysregulation, especially under stress, pain, or negative emotional stimuli.

#### 3.1.2. Detection Tools

Differences in facial expressions discovered in the aforementioned exploratory studies between cognitively impaired patients and healthy subjects are used in detection studies to distinctly identify cognitive impairments. Various studies have been conducted to perform such classifications of cognitively impaired patients and healthy controls using facial features, with psychiatric test scores used as ground truth (Table 1). These studies have focused on detecting AD, MCI, and dementia patient groups classified based on MMSE, MoCA, and other cognitive tests. Based on extracted facial features or complete facial images in facial videos classified with machine learning techniques, these studies reported higher predictive performances, demonstrating the feasibility of using facial expressions in the automated screening of cognitive impairments.

#### 3.1.3. Limitations

Although the above-listed studies report higher accuracies in detecting cognitive impairments, several limitations remain. One common limitation across all the studies is that they have used cross-validation with the same dataset; hence, they lack external validation. For example, the aforementioned studies primarily used data from participants from a specific ethnicity, but have not explored the generalizability of the reported accuracies to other ethnicities. Future research should employ multiple diverse datasets for training and testing to better assess model generalizability.

Additionally, it is required to evaluate the clinical reference of the reported models and their accuracy. While modern ML models are efficient in model performance, their feature explainability is often limited. Therefore, it would be necessary to develop explainable ML models that can clearly depict the clinical relevance of their significant features in the detection of cognitive impairments. This can help medical professionals gain insights into understanding the underlying features driving the models’ performance and enhance trust in their clinical applicability.

### 3.2. Facial Expressions in Pain Assessment

Pain is an unpleasant sensory and subjective emotional experience associated with actual or potential tissue or nerve damage, where individuals feel pain in different ways, even if the sources of pain are the same [51]. Depending on the duration and frequency of pain, it can take three primary patterns: (1) short duration ‘acute pain’, which can start and end suddenly; (2) ‘episodic pain’, which can occur time to time with regular or irregular intervals; and (3) ‘chronic pain’, which can last for more than three months. Additionally, pain is categorized based on its source: (1) ‘nociceptive pain’, caused by tissue damage or inflammation; (2) ‘neuropathic pain’, caused by nerve damage due to injury or disease; and (3) ‘nociplastic pain’, which describes the pain caused by changes in how the nervous system processes pain [52,53].

Due to its subjective nature in perception among individuals, self-reporting is considered the primary method of pain assessment [54]. These include the verbal rating scale (VRS), where subjects verbally rate their pain on a scale, the visual analog scale (VAS), where subjects mark their pain on a 100 mm visual scale [55], and the faces scale for reporting pain in children [56]. Despite its popularity and consideration as the ‘gold standard’ in pain assessment, these subjectively reported scales carry several limitations, including their lesser feasibility in cognitively impaired, unconscious, and non-verbal subjects. Therefore, objective measurements of pain have become important.

Multiple behavioral changes occur during pain experiences, including posture changes [57], vocalizations [58], and facial expression changes [59]. Pain evokes a ‘universal facial expression’ where a consistent set of facial AUs is activated across different painful stimuli [60]. Due to the observed changes in facial expressions during pain experiences, several studies have attempted to detect pain in subjects by studying their facial expressions. These studies are reported below (Table 2), with varying accuracies using ML-based techniques for pain detection and pain intensity estimation. One outcome from these research works is the introduction of a Prkachin and Solomon pain intensity score (PSPI). PSPI is based on a linear additive scoring of four AUs, i.e., brow lowering (AU04), orbit tightening (AU06 and AU07), upper-lip raising/nose wrinkling (AU9 and AU10), and eye closing (AU43) [61]. However, depending on the tool used for the detection of these AU activations (manual or automatic tools), when computing PSPI, it can produce inconsistencies in repeatability; hence, it could have limited applicability in clinical settings.

The majority of the above work on pain detection and pain intensity estimation has used the publicly available UNBC–McMaster database, which contains self-reported shoulder pain data, and the MIntPAIN database, which includes subjects experiencing evoked pain using electrical stimulation. Although most of these studies focused on cognitively intact patients capable of self-reporting their pain, several studies involving cognitively impaired patients have shown that their facial expressions reflect pain as effectively as, or even more clearly than, those of healthy controls [77,78]. These findings support the feasibility of automated objective pain assessment using facial expressions in healthy subjects as well as in cognitively impaired individuals. Consistent with this concept, a recent study has developed a commercial application called ‘PainCheck’ [79], which uses deep learning methods (i.e., automated facial recognition and analysis) to identify facial micro-expressions for the detection of the presence of pain, particularly aiming for pain assessment in people living with dementia.

#### 3.2.1. Limitations

Similarly to the studies reported on the detection of cognitive impairments using facial expressions (Table 1), the majority of the studies on pain reported above (Table 2) have used cross-validation within the same dataset to measure the performance, thus lacking generalizability. In contrast, Fontaine et al. (2022) [71] have reported their accuracy on an external dataset, while Alghamdi and Alaghband (2022) [72] and Tan et al. (2025) [74] have reported their accuracy on test data from unseen subjects. However, the majority of the studies have used data from two publicly available datasets with limited homogeneous subjects; therefore, further attention should be paid to potential inflation of the reported accuracies and the risk of over-fitting. Hence, the robustness of the reported accuracies should be evaluated with additional diverse datasets. Additionally, it would be necessary to develop explainable ML models, which can clearly demonstrate the relevance of the significant ML model features with clinical interpretability.

#### 3.2.2. Studies on Evaluating the Genuineness of Pain Expression

Apart from the above-reported studies focusing on pain vs. no-pain detection, there are a few studies focused on detecting genuine and fake pain using facial expressions. One such study, conducted with healthy subjects in which pain was evoked using a cold-pressure task (CPT), reported an accuracy of 85% in distinguishing genuine from fake pain using a support vector machine (SVM) classifier, compared to only 55% accuracy achieved by trained human observers [80]. In another similar study involving 26 participants undergoing pain evoked with CPT, a machine learning based classifier achieved an accuracy of 88% based on extracted AU facial features, whereas human performance reached only 49% accuracy [81]. These studies demonstrate the capabilities of machine learning-based methods in outperforming humans in fake vs. genuine pain detection and in overall pain intensity estimation.

### 3.3. Facial Expressions in Other Health Assessments

Beyond the changes in facial expressions associated with cognitive decline and pain perception, we also identified studies indicating that various other health conditions can also evoke measurable alterations in facial expressions in subjects compared to baseline conditions. These include chest pain and cardiac diseases, stroke, non-psychotic mental disorders, migraine, and infections, and they will be discussed below under different sections.

#### 3.3.1. Chest Pain and Cardiac Diseases

A recent study adopted deep learning models (YOLO) with higher accuracies (80–100%) to carry out real-time identification of chest pain conditions to assist the clinician–patient consultations and to reduce the extent of cardiac damage in patients. The study was carried out with data taken from videos of 1000 patients experiencing chest pain symptoms, and the reported accuracies are on a hold-back test dataset, which was 15% of the full dataset [84]. A recent study by Khedkar, R. et al. (2024), further confirmed the feasibility of using facial features in detecting cardiac diseases. The researchers demonstrated that a machine learning-based model was able to achieve an accuracy of 88% (for the test dataset) in predicting real-time coronary artery heart disease (CAD) through facial features extracted from facial images with characteristics commonly associated with CAD. They used 200 facial photographs, each from individuals diagnosed with CAD and an equal number from individuals without known cardiac conditions [85].

Chest pain can result from life-threatening events such as a myocardial infarction. Efficient and early detection of chest pain is therefore critical, as it can reduce the risk of severe or potentially fatal outcomes. Dalton et al. (1999), focused on discovering the facial expressions exhibited by 278 patients admitted to the emergency department with chest pain who were given a possible diagnosis of acute ischemic heart disease (AIHD). By analyzing the videotapes using FACS, they identified four AUs (lowering the brow (AU04), parting the lips (AU25), pressing the lips (AU24), and turning the head left (AU51)) that showed significant association with positive creatine kinase (CK) enzyme levels, a known biomarker and predictor of acute AIHD [82]. Another study conducted in an emergency department with 50 patients presenting with dyspnoea and chest pain, classified into disease+ (patients with serious diseases diagnosed with cardiopulmonary diagnosis) and disease- (patients who were well on telephone follow-up with no serious diagnosis) groups, found that disease+ group exhibited lower facial expression variability and surprise effect compared to the disease- group, when the stimuli-evoked facial expressions were analyzed with FACS scores [83].

#### 3.3.2. Stroke

Stroke is a medical emergency that requires prompt detection and treatment to reduce the damage to brain cells. It occurs when blood flow to part of the brain is interrupted (ischemic) or when a blood vessel ruptures (hemorrhagic). Facial expression changes are among the most visible and important signs during a stroke, such as drooping of the face on one side. Therefore, tracking the changes in facial features is an important method to detect the onset of a stroke.

Several studies have explored an automated approach focusing on these characteristics. Focusing on the facial expressional asymmetry and mouth skew of stroke patients, a previous study proposed the use of asymmetry indices (area ratio and distance ratio between the left and right side of the eye and mouth) to classify stroke patients with higher accuracies (with a total of 69 images for training and testing; Accuracies: 100% with SVM, 95.45% with Random Forest and 100% with Bayes) [86]. A similar study calculated facial features such as wrinkles on the forehead area, eye movement, mouth drooping, and cheek line detection to identify early symptoms of stroke using a testing dataset of 100 images and achieved an accuracy of 91% [87]. Since these higher accuracies are reported with only a limited number of facial images, further research is required to explore the generalizability of these proposed models.

With 3050 face images from stroke patients and normal people (75% for training and 25% for testing), Mohamed et al. (2025) reported an accuracy of 98.43% in detecting stroke patients. They carried out face detection using the YOLO8 model, followed by facial feature extraction and feature selection using active appearance model (AAM) and binary booby bird optimization (B3O), and finally a Naive Bayes (NB) classifier to detect stroke patients [88]. Another study involving 185 patients with acute ischemic stroke and 551 age and sex matched healthy controls reported an area under the curve (AUC) of 0.91 for stroke recognition based on facial images using an ensemble convolutional neural network (CNN) based model [89]. These studies demonstrated the applicability and feasibility of the facial expression-based approach for stroke detection.

#### 3.3.3. Non-Psychotic Mental Disorders

Non-psychotic mental disorders, which are typically less severe than psychotic disorders, affect an individual’s emotions and behavior without causing psychosis, such as delusions or hallucinations. Some examples are depression, Obsessive–Compulsive Disorder (OCD), Autism Spectrum Disorder (ASD), and Borderline Personality Disorder (BPD). Overall, multiple studies have shown that individuals with non-psychotic disorders exhibit attenuated facial expressions in response to emotional or sensory stimuli [90].

OCD, which is characterized by recurrent thoughts or obsessions and compulsive activities, is often underdiagnosed regardless of its worldwide spread [91]. A study conducted with 10 OCD patients and 10 healthy controls by using FACS to analyze emotional expression in response to film clips showed reduced congruent emotional expression in the OCD group compared to healthy controls [92]. Similar results were observed in a study with both OCD and mild OCD patients in comparison to healthy controls [93]. Depression is another example of non-psychotic disorders characterized by persistent feelings of sadness and reduced interest. Consequently, the expression of positive emotions is often impaired in depressed patients. This has been demonstrated in studies that used emotional stimuli, such as pictures or film clips, and were analyzed using EMFACS. These studies consistently reported reduced positive expressions in terms of both frequency and intensity [94,95]. Similarly, BPD patients exhibited reduced positive expressions and also diminished negative expressions compared to healthy controls when analyzed with EMFACS in response to film clips [94]. In ASD patients, reduced positive and negative dynamic expressions were also observed in response to film clips analyzed using FACS [96]. Collectively, these findings highlight that individuals with non-psychotic disorders display impaired emotional expression in response to stimuli, suggesting potential applications for these facial markers in both detection and diagnostic evaluation of such disorders.

#### 3.3.4. Migraine

Migraine, characterized by recurrent painful headaches, globally affects more than one billion people. A recent study analyzed changes in facial activity in individuals with migraine under calm and resting conditions using camera-based recordings. The study included 46 healthy subjects, 174 patients with episodic migraine (EM), and 84 patients with chronic migraine (CM). They found that the lid-tightener action unit was a reliable indicator of headache intensity [97], denoting the potential of facial feature-based approaches for migraine detection.

#### 3.3.5. Infections

Although the human immune system has evolved to adapt to the threats of infections, the probability of being infected is still high and can potentially cause serious complications; hence, the early detection of infections is important. Using an experimental model of sickness with 22 volunteers intravenously injected with either endotoxin (lipopolysaccharide; 2 ng/kg body weight) or placebo, a study found that the faces of subjects injected with endotoxin (after two hours of the injection) were perceived as more sick and less healthy. They also expressed more negative emotions (sadness and disgust) and less happiness and surprise, demonstrating the feasibility of using emotional expressions to detect infectious individuals [98].

### 3.4. Overall Comparison of Facial Expressions in Aforementioned Health Assessments

Facial expressions were found to deviate from healthy baselines in all of the aforementioned conditions. However, these deviations were specific to each assessed health condition. More specifically, some previous work reports that the expressions of emotions were reduced, leading to hypomimia, while others report the specific increase in the expression of negative affect in cognitive impairments. In comparison, condition-specific activations of facial muscles (AUs) were strongly associated with pain perception, stroke, migraine, and CAD, demonstrating the importance of analyzing individual muscle activation rather than interpreting the emotions conveyed by the face as a whole. In contrast, using facial expressions in the initial detection of non-psychotic disorders is less feasible, as condition-specific external stimuli are often required to elicit the facial expression deviations in these patients.

Hence, facial feature-based models that emphasize the disease-specific feature activations should be developed in the detection of the aforementioned health conditions, rather than relying on a generic facial expression-based analysis.

## 4. Discussion

In the present review, we have explored various facial expression analysis tools and their applicability in health assessment. As discussed in the above sections, facial expressions can be used in screening and detection of several health conditions, including cognitive impairments, pain assessment, stroke, migraine, and a multitude of other disorders. With the current advancements in computer vision and machine learning techniques, automated facial expression analysis has become efficient and fast in computation [99]; therefore, the applicability of this technique in healthcare settings has become extremely feasible. In addition to the computation efficiency, automated facial expression-based health monitoring carries several advantages due to its non-invasive nature. Unlike traditional brain scans or blood tests, which can be costly, resource-consuming, and overwhelming for patients, these techniques utilize facial video data and extracted facial features, which can be easily captured during routine interactions and interviews with healthcare professionals.

Most studies discussed in this review have used facial images or videos in controlled, optimum conditions; however, they have not evaluated the challenges in the real-world deployment of the introduced models. For example, in practice, lighting conditions, background complexity, and image and camera quality can affect the performance and accuracy of facial expression-based health assessment. Therefore, extended research should be conducted to evaluate the performance under diverse backgrounds and facial video quality settings. Additionally, extra steps should be employed to protect the patient’s privacy in recorded facial images and videos.

Another common drawback of these current studies lies in the selection of the ground truth parameter used to train the computational models. For example, studies related to cognitive decline have used psychiatric tests as ground truth, which themselves can carry several limitations (e.g., they are not strong enough to detect mild cognitive conditions and have a ceiling effect with high IQ patients). Pain assessment studies have used self-reported pain scores as the ground truth, which are subjective. Hence, the selection of more objective parameters as the reference in model development can significantly improve the objective health assessment nature of the studies. These could include vital signs (e.g., heart rate, blood pressure, etc.) and brain signals (e.g., EEG) that can potentially co-vary with facial expression changes in different health conditions. Therefore, incorporating further improvements in the proposed technologies can ensure their accuracy, usability, and efficiency in applying them in healthcare settings as an assisting tool for healthcare professionals.

From a clinical perspective, longitudinal assessment of health conditions is important. Therefore, evaluating the proposed models for their accuracy and compatibility in long-term patient monitoring over time would bring value to this technology. Additionally, since vital signals and other bio-signals are continuously monitored in the majority of clinical settings, it would be interesting to explore the expandability of proposed technologies to include multimodal fusion architectures. This could bring more disease-specific as well as patient-specific information to the facial expression-based technology and potentially improve the prediction reliability and accuracy. In the long term, it is important to identify the limitations and challenges of applying these technologies in hospital settings and in remote patient monitoring. These include navigating the regulatory pathways, obtaining medical certifications required for integration into existing clinical workflows, and conducting comparisons with established standard benchmarks. Most importantly, attention should be paid to safeguarding patient privacy when using facial videos (high privacy risk due to direct identification) and processed facial features (relatively low privacy risk). These include secure data storage practices, as well as controlling access rights to data.

All the discussed research work in the present article is tested with only subject groups of a homogenous ethnicity. However, it has been shown that facial emotion expressivity can change based on demographics, including gender [100,101], race, and age groups [102]. Therefore, to develop robust facial expression-based health assessment technologies, it is important to expand the studies by incorporating participants from different demographics. In addition, recent studies have shown the capabilities in capturing and recognizing facial micro-expressions in facial videos [103,104]. However, further research is required to evaluate the capabilities of capturing facial micro movements in clinical contexts when expanding these studies into real-world clinical settings.

Overall, the technologies developed in the studies reviewed here can be integrated into the existing healthcare system to assist healthcare professionals in making accurate and objective assessments of health conditions (with additional improvements to address the limitations in real-world deployment). Most importantly, this approach can be deployed in telemedicine applications to serve patients in remote areas with limited access to healthcare services and professionals. The real-world value of these technologies lies in their capabilities for early health screening in both onsite and remote settings, thereby enhancing the quality of care for individuals while helping to alleviate the growing demand on the current healthcare system.

## 5. Conclusions

In this review, we examined the application of facial expression analysis for detecting various health conditions, including cognitive impairments, pain, stroke, migraine, and several other disorders. Our findings indicate that each condition produces distinct deviations in facial features; therefore, disease-specific facial feature models that emphasize condition-relevant feature activations are necessary, rather than relying on generic facial expression-based analyses. Although the majority of studies reviewed report high accuracy in detecting different health conditions, several limitations remain. These include concerns related to patient privacy when using facial videos or images, challenges associated with meeting clinical standards for integration into existing workflows, and issues related to camera and image quality during clinical deployment. With appropriate improvements to address these limitations, facial expression-based technologies for disease detection can be deployed in both remote and onsite settings to enable objective health assessment. Once refined, these technologies have the potential to assist healthcare professionals with emergency triage, early diagnosis, and treatment planning, thereby helping to reduce the current excessive demand on healthcare systems.

## Figures and Tables

**Table 1:** Studies using facial expressions in detecting cognitive impairments.

Citation	Subject Population (Data Collection Setting)	Facial Features	Ground Truth	Classification Method (Validation Method)	Prediction Performance
Tanaka et al., 2019 [41]	12 dementia, 12 healthy subjects (human–agent integration videos)	Two-dimensional facial landmarks, face pose, gaze angles, AUs, lip movements	MMSE	L1 regularized logistic regression (cross-validation)	AUC of ROC 0.82
Umeda-Kameyama et al., 2021 [42]	121 CI, 117 healthy subjects (front-on portrait images of participants/no stimuli)	Facial images	MMSE	Multiple Deep learning models (e.g., Xception, SENet50, ResNet50, VGG16) (cross validation)	Accuracy 92.56% AUC of ROC 0.9717
Jiang et al., 2022 [43]	256 CI, 237 healthy subjects (passive viewing memory test)	Facial images	MoCA	CNN framework proposed in Jiang et al., 2021 [44] (cross-validation)	AUC of ROC 0.609
Fei et al., 2022 [45]	36 CI, 25 healthy subjects (under evoked emotions with video stimuli)	Frames from facial videos	MoCA	DNN (MobileNet and SVM) (cross-validation)	Accuracy 73.3%
Zheng et al., 2023 [46]	117 total subjects (CI and healthy subjects) (patient video during interviews)	AUs, Face mesh, HOG	MMSE	Deep learning-based system (SVM, LSTM) (cross-validation)	Accuracy with AUs 71%, Face mesh 66%, HOG 79%
Alsuhaibani et al., 2024 [47]	68 total subjects (MCI and healthy subjects) (video-recorded conversations at home)	Facial images from facial videos	Clinical diagnosis	Deep learning-based framework (cross-validation)	Accuracy 88%
Sun et al., 2024 [48]	100 MCI, 89 healthy subjects (video chats from controlled behavioral intervention)	Facial video clips	Clinical diagnosis	Transformer-based framework (cross-validation)	Accuracy 90.63%
Takeshige-Amano et al., 2024 [49]	93 AD, 99 healthy subjects (natural conversations with a chatbot)	Smile, face orientation, eye opening, and blink indices	MMSE, MoCA	Multiple Machine learning classifiers (e.g., Random Forest, logistic regression) (cross-validation)	Accuracy 0.72 (with Random Forest classifier)
Okunishi et al., 2025 [50]	110 MCI, 144 Dementia, 161 healthy subjects (patient video during interviews)	AUs, emotion categories, Valence-Arousal, face embeddings	MMSE	Decision tree-based model (Light GBM) (cross-validation)	AUC of ROC dementia: 0.933, MCI: 0.889

CI: Cognitive impaired.

**Table 2:** Studies using facial expressions in detecting pain and estimating pain intensities.

Citation	Subject Population	Facial Features	Ground Truth	Classification Method (Validation Method)	Prediction Performance
Lucey et al., 2011 [62]	25 subjects with shoulder pain ^1^	Facial video frames (active appearance model (AAM)-based system for feature extraction)	PSPI	SVM (pain vs. no-pain) (cross-validation)	Accuracy 80.9%
Rathee, Ganotra, 2015 [63]	25 subjects with shoulder pain ^1^	Facial video frames	PSPI	Distance Metric Learning (DML) + SVM for 16-level pain intensity classification (Cross-validation)	Accuracy 96%
Rathee, Ganotra, 2016 [64]	25 subjects with shoulder pain ^1^	Facial video frames (extracted Gabor, HOG, and local binary pattern features)	PSPI	Multiview DML + SVM for pain detection and 4-level pain intensity classification (cross-validation)	Accuracy for Pain detection: 89.59% Pain intensity: 75%
Bargshdy et al., 2020a [65]	25 subjects with shoulder pain ^1^	Facial video frames	PSPI	Deep learning-based framework for 4-level pain intensity classification (cross-validation)	Accuracy 85%
Bargshdy et al., 2020c [66]	25 subjects with shoulder pain ^1^, 20 subjects with electrically evoked pain ^2^	Facial video frames in Hue, Saturation, Value (HSV) color space	PSPI and stimuli-based pain scale	Temporal CNN for 4-level pain intensity classification for Dataset 1 and 5-level pain intensity for Dataset2 (cross-validation)	Accuracy for Dataset1: 94.14% Dataset2: 89%
Bargshdy et al., 2020b [67]	25 subjects with shoulder pain ^1^, 20 subjects with electrically evoked pain ^2^	Facial video frames	PSPI and stimuli-based pain scale	CNN-RNN for 5-level pain intensity classification for Dataset 1 and 5-level pain intensity for Dataset2 (cross-validation)	Accuracy for Dataset1: 86% Dataset2: 92.26%
Casti et al., 2021 [68]	25 subjects with shoulder pain ^1^	Facial video frames	VAS	Linear discriminant analysis (LDA) for pain detection (VAS > 0 vs. VAS = 0) and Pain intensity (VAS) estimation (cross-validation)	Accuracy for Pain detection AUC 0.87 Pain intensity estimation MAE 2.44
Barua et al., 2022 [69]	129 subjects with shoulder pain ^1^	Facial video frames (shutter blinds-based deep feature extraction)	PSPI	kNN for 4-level pain intensity classification (cross-validation)	Accuracy 95.57%
Rodriguez et al., 2022 [70]	25 subjects with shoulder pain ^1^	Facial video frames	PSPI	CNN-LSTM-based method for pain detection and 6-level pain intensity estimation (cross-validation)	Accuracy of pain detection: 83.1% pain intensity estimation: MAE 0.5
Fontaine et al., 2022 [71]	1189 patients before and after surgery	Facial images and AUs	NRS	CNN-based pain intensity estimation for facial images and SVM for AUs (external test dataset)	Accuracy CNN 53% SVM 27.7%
Alghamdi, Alaghband, 2022 [72]	25 subjects with shoulder pain ^1^ (24 for model development, 1 for unseen test data)	Facial video frames	PSPI	Transfer learning-based approach (inceptionV3 with SGD optimizer) for 4-level pain intensity classification (cross-validation and 1 unseen subject)	Accuracy 90.56% on unseen subject data; 99.10% on 10-fold CV
Alphonse et al., 2024 [73]	25 subjects with shoulder pain ^1^	Facial video frames (Statistical Frei-Chen Mask (SFCM)-based features and DenseNet-based features)	PSPI	Radial Basis Function-Based Extreme Learning Machine (RBF-ELM) classifier for 4-level pain intensity estimation (cross-validation)	Accuracy for pain intensity estimation 98.58%
Tan et al., 2025 [74]	200 patients undergoing surgery or interventional pain procedures (160 subjects for training, 40 for validation)	Facial video frames	NRS	Spatial-temporal attention long short-term memory (STA-LSTM) deep learning network for 3-level pain intensity (40 unseen subjects for validation)	Accuracy 86.6%

Datasets: ^1^ UNBC–McMaster database [75] contains videos of the faces of adult subjects with a rotator cuff and other shoulder injuries. The subjects were recorded during the movement of their affected and unaffected shoulders during active (where the subject moves the arm himself) and passive (where the subject’s arm is moved by a physiotherapist) conditions. ^2^ MIntPAIN database [76], which was captured by giving electrical muscle pain stimulation to the subjects.

## Data Availability

No new data were created or analyzed in this study.
